# Assessing the National Prevalence of HIV Screening in the United States using Electronic Health Record Data

**DOI:** 10.7759/cureus.5043

**Published:** 2019-06-30

**Authors:** Joshua D Niforatos, Jonathon W Wanta, Emily Durbak, Jacqueline Cavendish, Justin A Yax

**Affiliations:** 1 Emergency Medicine, Cleveland Clinic Lerner College of Medicine, Case Western Reserve University, Cleveland, USA; 2 Psychiatry and Behavioral Sciences, University of Washington, Seattle, USA; 3 Miscellaneous, Case Western Reserve University, Cleveland Clinic Lerner College of Medicine, Cleveland, USA; 4 Emergency Medicine, University Hospitals Cleveland Medical Center, Cleveland, USA; 5 Emergency Medicine and Internal Medicine, University Hospitals Cleveland Medical Center, Cleveland, USA

**Keywords:** hiv screening, electronic health record, health care technology

## Abstract

The Centers for Disease Control and Prevention and the U.S. Preventive Services Task Force recommend population-based screening for human immunodeficiency virus (HIV) at least once in each patient's life. National surveys estimate that 42.5% of the population has been screened; however, these studies have relatively low sample sizes and inherent survey biases. Using a national, de-identified cloud-based electronic health record (EHR) information from over 48 million patients, we found that only 6.4% of Americans over the age of 18 had laboratory evidence of a prior HIV test. Further investigation is necessary to determine if single-item questions on national surveys correlate with objective evidence of HIV testing, as well as addressing the numerous limitations related to the use of EHR data that likely grossly underestimates the prevalence of HIV screening nationally.

## Introduction

Population-based screening for human immunodeficiency virus (HIV) is recommended by both the Centers for Disease Control and Prevention (CDC) and the United States Preventive Services Task Force [1-2]. The CDC estimates 42.5% of the US population of 18 years of age and older has been screened for HIV [3]. National, question-based surveys provide data for this prevalence estimate [4]. We sought to estimate the prevalence of HIV screening in the United States using laboratory data from real-time Electronic Health Record (EHR) data of over 60 million unique patients over 18 years.

## Materials and methods

We utilized the cloud-based Explorys, Inc. (Cleveland, OH) database. De-identified and standardized aggregate data from 60 million patients are uploaded daily to Explorys from 26 integrated-US healthcare systems across all 50 states. An in-depth description of the methodology and technical features of Explorys has been previously described in the literature [5], and has been validated across numerous fields, including dermatology, endocrinology, neurology, gynecology, gastroenterology, orthopedics, surgery, and hematology [6-13]. Briefly, data from EHRs is mapped onto the unified medical language system (UMLS) that is standardized and normalized, namely, the Systematized Nomenclature of Medical Clinic Terms for clinical term (SNOMED-CT) hierarchies, allowing researchers to utilize the web application’s PopEx system to search for disease, procedures, and laboratory results at the epidemiological level of a de-identified, aggregate patient cohort. SNOMED-CT is akin to the Clinical Classification Software (CCS) codes used to analyze data from the Agency for Healthcare Research and Quality. Use of Explorys has been deemed exempt from institutional review board approval by University Hospitals Cleveland Medical Center.

Data were collected from 1999 to April 3, 2018. We included all non-deceased patients over the age of 18 years, and excluded all patients with the ICD-9 diagnostic code for human immunodeficiency virus infection (042). For those who met the inclusion criteria, demographic data and history of HIV screening were collected. HIV screening included: second generation IgG anti HIV-1, IgG anti HIV-2, HIV-1 western blot (WB), HIV-1 immunofluorescence (IFA), HIV-2 enzyme-linked immunosorbent assay and WB; third generation IgG and IgM anti HIV-1, HIV-2, Group 0, HIV-1 WB or IFA, HIV-2 ELISA and WB; fourth generation IgG and IgM anti HIV-1, HIV-2, Group 0, HIV-1 p24 antigen, HIV-1,2 differentiation assay, HIV-1 RNA PCR; fifth generation IgG and IgM anti HIV-1, HIV-2, Group 0, HIV-1 p24 antigen, HIV-1 antigen and antibody, HIV-2 antigen and antibody [14].

We compared this data to the entire Explorys population over the age of 18 years with no previous history of HIV infection. Demographic data are presented as numbers and percentages. Prevalence of HIV screening was age-adjusted for sex comparisons, sex-adjusted for age-group comparisons, and sex- and age-adjusted for race comparisons. χ2 Tests and multiple pairwise comparisons with Bonferroni correction were used to assess differences between groups. Logistic regression was performed to model the effect of age, sex, race, and insurance status on HIV screening. The two one-sided t test (TOST) with +/- 10 point margin was used to assess the equivalence of group prevalence estimates between Explorys and CDC data as recommended by Tatem et al. [15]. Statistical significance was set to p < 0.05. All analyses were performed in either IBM SPSS Statistics, Version 25 (IBM) or Microsoft Excel, Version 16.11.1 with χLSTAT software for equivalence testing.

## Results

Table 1 lists characteristics of patients screened for HIV in the Explorys population. We identified 45,536,480 unique patients, of which 2,925,320 (6.4%) have laboratory evidence of HIV screening. Females (7.9%), African Americans (10.3%), 25-34-year olds were more likely to be tested for HIV in the Explorys population (p < 0.0005). Equivalence testing was not statistically significant (p > 0.98) for all group comparisons between Explorys and CDC data. However, both data sources share similar sex-, age-, and race-adjusted distributions trends in the testing (Table 1). Among patients screened for HIV, a multivariable logistic regression model showed that insurance status was the most important predictor of screening (Medicare; adjusted odds ratio [AOR], 3.1; 95% CI, 3.07-3.14; p < 0.0005). Other independent predictors included sex (female; AOR, 1.82; 95% CI, 1.81-1.824), age (< 40; AOR, 2.27; 95% CI, 2.25-2.29), and self-pay status (AOR, 1.95; 95% CI, 1.93-1.96).

**Table 1:** Adjusted Group-Specific Prevalence for HIV Screening in the United States Among Patients Over 18 Years and Older Abbreviations: HIV, Human Immunodeficiency Virus a All global χ2 tests were significant with a p < 0.0005 b All pairwise comparisons between subgroups were significant with a p < 0.0005. c All 95% CI using the Wilson Score method were within +/- 0.001 to +/- 0.05 of the prevalence for all calculations. d Nationally estimated prevalences from the National Health Interview Survey, 1997 - September 2017, Sample Adult Core Component. e Equivalency testing was not statistically significant (p > 0.98) at +/- 10.0 percentage points for all groups. Explorys, Inc. f "Other" races not included in this study.

Characteristics	No. Screened for HIV	Population Size	Prevalence, % ^a,b,c^	National Estimates of Prevalence, % (95%CI)^d,e^
Gender				
Male	942,960	20,311,900	4.6	42.3 (40.52–44.08)
Female	1,982,360	25,224,580	7.9	50.6 (48.86–52.32)
Race/Ethnicity^f^				
Caucasian	1,810,250	26,209,030	6.9	38.7 (37.45–40.02)
Black	480,000	4,678,880	10.3	61.1 (58.25–63.94)
Hispanic	210,680	2,186,000	9.6	47.2 (44.30–50.15)
Age				
18-24	208,480	4,011,290	5.2	31.9 (29.14-34.72
25-34	852,490	7,511,680	11.3	54.6 (52.31-56.89)
35-44	759,030	7,340,790	10.3	56.8 (54.39-59.16)
45-64	914,510	14,769,970	6.2	42.5 (40.85-44.13)
65 and over	190,810	11,902,750	1.6	19.1 (17.46-20.78)
Overall	2,925,320	45,536,480	6.4	42.5 (41.42–43.55)

## Discussion

We sought to eliminate biases associated with survey questions by characterizing the prevalence of HIV screening in the United States using laboratory data from one of the largest, nationally distributed patient population databases. A preliminary analysis identified the prevalence of people living with HIV in Explorys to be 0.33%, which approximates the CDC reported a prevalence of 0.37% [16]. However, in this study, we identified only 6.4% of the Explorys population as ever-screened for HIV compared to 42.5% estimated by the CDC. Equivalence testing was non-significant indicating these databases are not equivalent for estimating HIV screening. While our estimates were significantly lower than those reported by the CDC, it should be noted that no study has yet determined whether single-item questions on national surveys of HIV screening corroborate with objective evidence of screening. Regardless, females, African Americans, and persons under 40 were more likely to be screened in the Explorys population, which corroborates the demographic distribution of screening observed in national surveys [3].

This study's limitations are important and relevant to the use of EHR data for HIV screening. First, this study was limited by hospitals systems that do not report HIV laboratory data to Explorys or use anonymous HIV screening. Second, information from patients who receive screening through non-hospital systems, such as county health departments, stand-alone STD clinics, are not included in the Explorys database. Third, the conversion from paper charts to EHRs for the included hospital systems may result in missing data since 1999; however, routine screening for HIV was not recommended by the CDC until 2006 and by the USPSTF until 2013, thus providing a lag time for routine uptake of this clinical practice as hospitals adopt the EHR. These limitations taken together suggest that use of the EHR for assessing the prevalence of HIV screening in the United States should not currently be utilized in health services research and are likely the reason for the significant discrepancy observed in this study. 

## Conclusions

It is estimated that the prevalence of population-based HIV screening in the United States relies on survey questions that may not be reliable and often represents data from the previous year or later. Although this study reveals the profound limitations with EHR data thus rendering it currently not useful for the study of HIV laboratory data, if these limitations can be addressed nationally, cloud-based all-payer databases may provide objective, daily up-to-date information on HIV screening daily. Until then, studies using EHR or administrative claims data should interpret HIV laboratory data with caution as it may greatly underestimate the proportion of patients screened in the United States.
